# Methylene Blue Prevents Retinal Damage Caused by Perinatal Asphyxia in the Rat

**DOI:** 10.3389/fncel.2020.00157

**Published:** 2020-06-04

**Authors:** Juan Carlos Fernández, Rafael Peláez, Manuel Rey-Funes, Manuel Soliño, Daniela S. Contartese, Verónica B. Dorfman, Juan José López-Costa, Ignacio M. Larrayoz, César F. Loidl, Alfredo Martínez

**Affiliations:** ^1^Instituto de Biología Celular y Neurociencia “Prof. E. de Robertis”, Facultad de Medicina, Universidad de Buenos Aires, Buenos Aires, Argentina; ^2^Primera Cátedra de Farmacología, Facultad de Medicina, Universidad de Buenos Aires, Buenos Aires, Argentina; ^3^Center for Biomedical Research of La Rioja (CIBIR), Logroño, Spain; ^4^Centro de Estudios Biomédicos, Biotecnológicos, Ambientales y Diagnóstico (CEBBAD), Universidad Maimónides, Buenos Aires, Argentina

**Keywords:** angiogenesis, apoptosis, electroretinography, inner retinal thickness, methylene blue, perinatal asphyxia

## Abstract

Perinatal asphyxia (PA) is responsible for a large proportion of neonatal deaths and numerous neurological sequelae, including visual dysfunction and blindness. In PA, the retina is exposed to ischemia/reoxygenation, which results in nitric oxide (NO) overproduction and neurotoxicity. We hypothesized that methylene blue (MB), a guanylyl cyclase inhibitor, and free-radical scavenger currently used in the clinic, may block this pathway and prevent PA-induced retinal degeneration. Male rat pups were subjected to an experimental model of PA. Four groups were studied: normally delivered (CTL), normally delivered treated with 2 mg Kg-1 MB (MB), exposed to PA for 20 min at 37°C (PA), and exposed to PA and, then, treated with MB (PA-MB). Scotopic electroretinography performed 45 days after birth showed that PA animals had significant defects in the a- and b-waves and oscillatory potentials (OP). The same animals presented a significant increase in the thickness of the inner retina and a large number of TUNEL-positive cells. All these physiological and morphological parameters were significantly prevented by the treatment with MB. Gene expression analysis demonstrated significant increases in iNOS, MMP9, and VEGF in the eyes of PA animals, which were prevented by MB treatment. In conclusion, MB regulates key players of inflammation, matrix remodeling, gliosis, and angiogenesis in the eye and could be used as a treatment to prevent the deleterious visual consequences of PA. Given its safety profile and low cost, MB may be used clinically in places where alternative treatments may be unavailable.

## Introduction

Perinatal asphyxia (PA) is the most severe perinatological problem across the world (World Health Organization, 1991) and is associated with approximately one-quarter of global neonatal deaths (Liu et al., 2015). PA generates a transient global ischemic status which could damage the central nervous system, including the retina (Ferriero, 2004). Depending on the length and intensity of the ischemic episode, PA sequelae may include attention-deficit hyperactivity disorder, spasticity, epilepsy, mental retardation (Herrera et al., 2018), and hearing or visual dysfunctions, including blindness (Hill, 1991). In 2010, there were an estimated 1.15 million cases of neonatal encephalopathy, of which 96% were from low- and middle-income countries (Mukhtar-Yola et al., 2018). Therapeutic hypothermia is currently the standard of care for newborns exposed to PA (Rivero-Arias et al., 2019), but this treatment may require expensive devices to be properly applied (Dingley et al., 2015) that could be prohibitive for some developing regions of the world. Our long-term goal is to investigate and promote the use of safe and affordable drugs in the prevention of visual loss associated with PA.

Exposure of the retina to hypoxia/ischemia-reoxygenation induces the expression of hypoxia-inducible factor-1α (HIF1α) and its target genes such as vascular endothelial growth factor (VEGF), adrenomedullin (AM), and inducible nitric oxide synthase (iNOS), among others (Rey-Funes et al., 2011). VEGF and AM are angiogenic factors that contribute to the thickening of the inner layers of the retina (Rey-Funes et al., 2013), whereas iNOS produces nitric oxide (NO) which reacts with the free radical superoxide resulting in elevated levels of peroxynitrites and extensive protein nitration, leading to neuronal cell death (Rodrigo et al., 2005). Excessive NO formation induces cytotoxic effects in the retina and is postulated as a key neurotoxic factor in retinal ischemia (Osborne et al., 2004). Therefore, inhibitors of NO activity may constitute valuable drugs for preventing retinal damage in the context of PA.

Most physiological actions of NO are mediated by the formation of the second messenger cGMP, which is produced by the action of guanylyl cyclase (Ding and Weinberg, 2007). Methylene blue (MB) is a guanylyl cyclase inhibitor (Hwang et al., 1998) which is also able to inhibit NADPH oxidase and myeloperoxidase enzymes (Heydrick et al., 2007) by either acting as a free radical scavenger or competing for oxygen (Atamna et al., 2008). MB exhibits a high safety profile (Bewick and Pfleiderer, 2014; Landoni et al., 2014) and is approved for clinical use as an antidote of poison-induced methemoglobinemia (Wright et al., 1999), in norepinephrine-refractory hypotension (Sparicio et al., 2004), and for the surgical management of hyperparathyroidism (Bewick and Pfleiderer, 2014), among others. MB is on the World Health Organization’s List of Essential Medicines, the most effective and safe medicines needed in a health system (World Health Organization, 2017). Also, MB has been shown to prevent retinal damage induced by rotenone (Zhang et al., 2006) or optic neuropathy (Rojas et al., 2009) in animal models. In a previous article, we showed that MB was able to significantly reduce morphological and molecular hallmarks of retinal damage caused by PA when applied preventively to the pregnant dams before delivery (Rey-Funes et al., 2016). The risks of suffering PA are well known (Martinez-Biarge et al., 2013) and the application of MB to high-risk mothers may reduce vision loss in their children. Nevertheless, we understand that an efficacious treatment that is applied after PA has been diagnosed would be better received by patients and the medical staff. In consequence, this report aimed to demonstrate the beneficial effects of treating asphyctic newborns with MB on physiological, morphological, and molecular markers of retinal damage.

## Materials and Methods

### PA Animal Model

Severe PA was induced using a noninvasive model of hypoxia-ischemia as described (Loidl et al., 2000). Sprague–Dawley albino rats with genetic quality and sanitary certification from the animal facility of our Institution were cared for following the guidelines published in the ARVO Statement for the Use of Animals in Ophthalmic and Vision Research. The procedures described below were approved by the Ethical Committee of CICUAL (Comité Institucional para el Uso y Cuidado de Animales de Laboratorio, Resolution N° 2079/07), Facultad de Medicina, Universidad de Buenos Aires, Argentina. Appropriate proceedings were performed to minimize the number of animals used and their suffering, pain, and discomfort. Animals were kept under standard laboratory conditions at 24°C, with light/dark cycles of 12/12 h, and food and water were provided *ad libitum*. Thirty timed-pregnant Sprague–Dawley rats were sacrificed by decapitation and immediately hysterectomized after their first pups were delivered vaginally. These normally delivered, non-manipulated pups were used as controls. Full-term fetuses, still inside the uterus, were subjected to asphyxia performed by transient immersion of both uterine horns in a water bath for 20 min at 37°C. After asphyxia, the uterine horns were opened, pups were removed, dried of delivery fluids, stimulated to breathe, and their umbilical cords were ligated. Pups were then placed for recovery under a heating lamp and given to surrogate mothers. To avoid the influence of hormonal variations due to the female estrous cycle, only male pups were included in this study. One hour after birth, control newborns were randomly divided into two groups, which received a 50 μl subcutaneous injection of either saline solution (CTL group, *n* = 30) or a dose of 2 mg Kg^−1^ methylene blue in saline solution (Sigma, St. Louis, MO, USA; MB group, *n* = 30). The same procedure was implemented with asphyctic newborns to generate the other two experimental groups: asphyctic animals that received saline (PA group, *n* = 30) or methylene blue treatment (PA-MB group, *n* = 30).

### Electroretinograms

Forty-five days after birth, young rats (*n* = 10 per experimental group) were subjected to scotopic electroretinography, as described (Rey-Funes et al., 2017). Briefly, after overnight adaptation in the dark, rats were anesthetized with 40 mg/Kg ketamine (Ketamine 50^®^, Holiday-Scott SA, Beccar, Argentina) + 5 mg/Kg xylazine (Kensol^®^, Laboratorios Köning SA, Buenos Aires, Argentina) under dim red illumination. An ophthalmic solution of 5% phenylephrine hydrochloride and 0.5% tropicamide (Fotorretin, Poen, Buenos Aires, Argentina) was used to dilate the pupils. Rats were placed facing the stimulus at a distance of 25 cm in a highly reflective environment. A reference electrode was placed through the ear, a grounding electrode was attached to the tail, and a gold electrode was placed in contact with the central cornea. Scotopic electroretinograms (ERG) were recorded from both eyes simultaneously and 20 responses were collected to flashes of unattenuated white light (1 ms, 1 Hz) from a photic stimulator (light-emitting diodes) set at maximum brightness. The registered response was amplified (9 cd s/m^2^ without filter), filtered (1.5-Hz low-pass filter, 500 Hz high-pass filter, notch activated), and averaged (Akonic BIO-PC, Buenos Aires, Argentina). The a-wave was measured as the difference in amplitude between the recording at onset and the trough of the negative deflection and the b-wave amplitude was measured from the trough of the a-wave to the peak of the b-wave. Values from each eye were averaged, and the resultant mean value was used to compute the group’s mean a- and b-wave amplitudes ± SEM. To calculate oscillatory potentials (OP), the same photic stimulator was used with filters of high (300 Hz) and low (100 Hz) frequency. The amplitudes of the OP were estimated by using the peak-to-trough method. The sum of three OP was used for statistical analysis.

### Tissue Processing, Histology, and TUNEL

Rats on the four experimental groups were sacrificed 6 days postpartum (*n* = 4 per experimental group). Animals were decapitated. After enucleating, anterior segments of the eyes, including the lens, were discarded, and the posterior segments of the eyes containing the retinas were fixed in 4% paraformaldehyde in 0.1 M pH 7.4 phosphate buffer at 4°C for 48 h. Tissues were dehydrated and paraffin-embedded. Tissue sections (5 μm-thick) were stained for terminal deoxynucleotidyl transferase dUTP nick end labeling (TUNEL) with the in situ Cell Death Detection POD Kit (Roche, Basel, Switzerland), following manufacturer’s instructions. Visualization of immunoreactivity was performed with 0.03% 3,3′diaminobenzidine (Sigma Co, St. Louis, MO, USA), 3% nickel ammonium sulphate and 0.01% hydrogen peroxide diluted in 0.1 M buffer acetate, yielding a black product.

The animals used for electroretinography were intraperitoneally anesthetized with ketamine/xylazine and intracardially perfused with the same fixative. The posterior segments of the eyes were paraffin-embedded, sectioned, and stained with hematoxylin-eosin to count the number of ganglion cells and to measure the thickness of the most inner layers of the retina (IR), which includes the internal limiting membrane, the retinal optic nerve fiber layer, and the ganglion cell layer (GCL), as reported (Rey-Funes et al., 2013).

### Immunofluorescence and Confocal Microscopy

Additional tissue sections were dewaxed, rehydrated, and subjected to antigen retrieval (10 mM sodium citrate, 0.5% Tween 20, pH 6.0, 30 min at 95°C). Non-specific binding was blocked by exposure to 10% normal donkey serum (Jackson Immunoresearch Laboratories, West Grove, PA, USA) for 30 min, and then tissue sections were incubated with primary antibodies (Table 1), overnight at 4°C. The following day, the presence of the primary antibody was detected by incubation with fluorescent secondary antibodies (Table 1) and counterstained with DAPI (Molecular Probes, Eugene, OR, USA). These slides were analyzed with a confocal microscope (TCS SP5, Leica, Badalona, Spain).

**Table 1:** Antibodies used in this study.

Primary antibodies				
**Target**	**Species**	**Dilution**	**Source**	**Reference**
GFAP	Rabbit polyclonal	1:500	Dako	Z0334
HNEJ-2	Mouse monoclonal	1:25	Abcam	ab48506
8-OHdG	Mouse monoclonal	1:50	Santa Cruz	sc-66036
**Secondary antibodies**				
**Specificity**	**Label**	**Dilution**	**Source**	**Reference**
Donkey anti-rabbit	Alexa Fluor^®^ 555	1:200	Invitrogen	A31572
Donkey anti-mouse	Alexa Fluor^®^ 488	1:600	Invitrogen	A21202

### Image Analysis

The eyes of four animals of each experimental group were analyzed. Care was taken on selecting anatomically matched areas of retina among animals before assays. The central area of the sagittal plane was chosen for each retina. The thickness of all layers in the retina was measured in 10 fields using Scion Image software. Special attention was paid to the inner retina (IR), which includes the internal limiting membrane, the optic retinal nerve fiber layer, and the GCL.

TUNEL-positive cells were counted on the retinas of four eyes per experimental group. Fields were chosen in the central region of retinal cross-sections and the number of positive cells in ten fields, 500 μm in length, was recorded.

### RNA Extraction and Quantitative Real-Time PCR

Animals from all experimental groups (*n* = 4 per experimental group) were sacrificed at different times after MB treatment (4, 6, 12, 24 h). The posterior chambers of the eyes were homogenized with TRIzol (Invitrogen, Madrid, Spain) and RNA was isolated with RNeasy Mini kit including a DNAse I on-column digestion (Qiagen, Germantown, MD, USA). One microgram of total RNA was reverse-transcribed into first-strand cDNA using random primers and the SuperScript III kit (Invitrogen) in a total volume of 20 μl according to the manufacturer’s instructions. Reverse transcriptase was omitted in control reactions, where the absence of PCR-amplified DNA confirmed the lack of contamination from genomic DNA. Resulting cDNA was mixed with SYBR Green PCR Master Mix (Applied Biosystems, Carlsbad, CA, USA) for quantitative real-time polymerase chain reaction (qRT-PCR) using 0.3 μM forward and reverse oligonucleotide primers (Table 2). Quantitative measures were performed using a 7300 Real-Time PCR System (Applied Biosystems). Cycling conditions were an initial denaturation at 95°C for 10 min, followed by 40 cycles of 95°C for 15 s and 60°C for 1 min. In the end, a dissociation curve was implemented from 60 to 95°C to validate amplicon specificity. Gene expression was calculated using relative quantification by interpolation into a standard curve. All values were divided by the expression of the housekeeping gene 18S.

**Table 2:** Primers used for quantitative real-time polymerase chain reaction (qRT-PCR) in this study.

Target gene	Forward primer	Reverse primer
iNOS	AGGCCACCTCGGATATCTCT	GCTTGTCTCTGGGTCCTCTG
IL1β	CCTCTGCCAAGTCAGGTCTC	GAATGTGCCACGGTTTTCTT
TNFα	GAGAGATTGGCTGCTGGAAC	TGGAGACCATGATGACCGTA
MMP2	ACCGTCGCCCATCATCAA	CCTTCAGCACAAAGAGGTTGC
MMP9	TGTCCAGACCAAGGGTACAGC	GAAGAATGATCTAAGCCCAGCG
GFAP	GAAGAAAACCGCATCACCAT	GGCACACCTCACATCACATC
VEGF	GCCAGCACATAGGAGAGATGAGC	CAAGGCTCACAGTGATTTTCTGG
PEDF	ACCCTCGCATAGACCTTCAG	GGCATTTCCCTTGTAGACCG
18S	ATGCTCTTAGCTGAGTGTCCCG	ATTCCTAGCTGCGGTATCCAGG

*The annealing temperature was 60°C for all primers. 18S was used as a housekeeping gene*.

### Statistical Analysis

All data were analyzed with GraphPad Prism 5 software and were considered statistically significant when *p* < 0.05. Values are expressed as means ± SEM. Normally distributed data were evaluated by ANOVA followed by either Holm-Sidak or Newman–Keuls *post hoc* test.

## Results

### MB Treatment Prevents Asphyxia-Induced Modifications of the a- and b-Waves and the Oscillatory Potentials of the Electroretinogram

**Figure 1:**
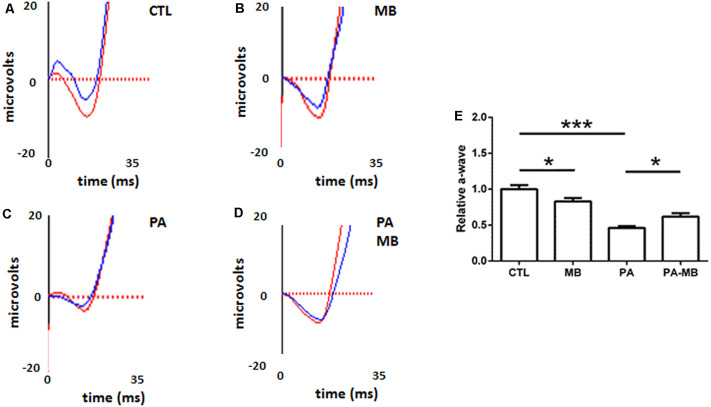
Representative scotopic electroretinograms (ERG) showing the a-waves on the four experimental groups: CTL **(A)**, methylene blue (MB; **B**), perinatal asphyxia (PA; **C**), and PA-MB **(D)**. The response of the right eye is represented by red lines and that of the left one by blue lines. Quantification of the a-wave amplitude, relativized to the CTL group, is represented as a histogram **(E)**. Bars represent the mean ± SEM of all samples (*n* = 10 animals per group). Asterisks represent statistically significant differences. *p < 0.05; ***p < 0.001. Statistical test: ANOVA followed by Holm-Sidak *post hoc* test.

**Figure 2:**
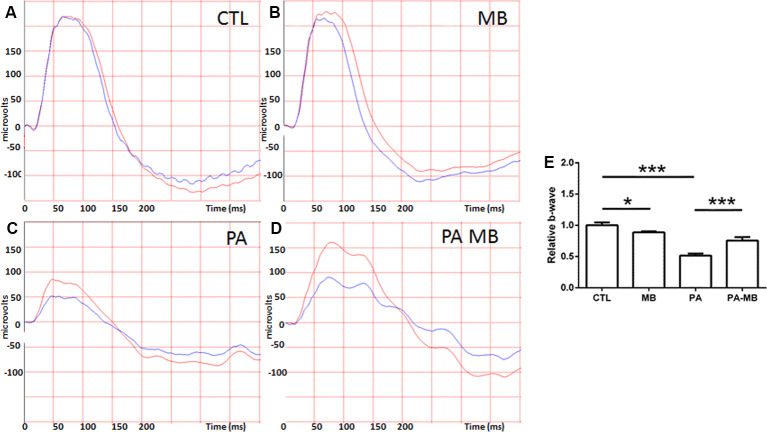
Representative scotopic ERG showing the b-waves on the four experimental groups: CTL **(A)**, MB **(B)**, PA **(C)**, and PA-MB **(D)**. The response of the right eye is represented by red lines and that of the left one by blue lines. Quantification of the b-wave amplitude, relativized to the CTL group, is represented as a histogram **(E)**. Bars represent the mean ± SEM of all samples (*n* = 10 animals per group). Asterisks represent statistically significant differences. *p < 0.05; ***p < 0.001. Statistical test: ANOVA followed by Holm-Sidak *post hoc* test.

Animals were divided into four experimental groups and treated as explained in the “Materials and Methods” section. The overall mortality rate for the asphyctic group was 40%, similar to previous reports (Loidl et al., 2000). Injection of MB, a well-known dye (Li et al., 2018), left a blue area under the skin of treated animals but this stain disappeared 2 or 3 days after injection. Scotopic ERG performed 45 days after birth showed that those animals that had suffered PA had a significant (*p* < 0.001) reduction of the a- and b-wave amplitude (Figures 1C,E, 2C,E) compared to those who were born uneventfully (Figures 1A,E, 2A,E). Treatment of the control animals with MB resulted in a mild reduction (*p* < 0.05) in the amplitude of both waves (Figure 1B,E, 2B,E). Notably, MB treatment of asphyctic pups resulted in a- (*p* < 0.05) and b- (*p* < 0.001) waves more similar to the controls than to the PA animals (Figures 1D,E, 2D,E).

**Figure 3:**
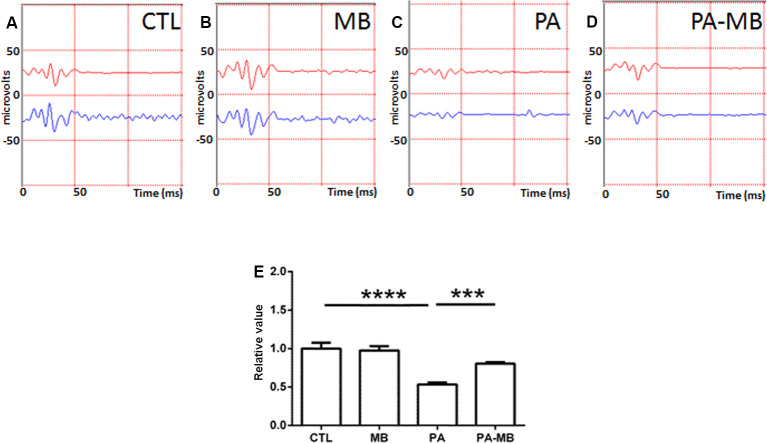
Representative scotopic ERG showing the oscillatory potentials (OP) on the four experimental groups: CTL **(A)**, MB **(B)**, PA **(C)**, and PA-MB **(D)**. The response of the right eye is represented by a red line and that of the left one by a blue line. Quantification of the relative OP sums, relativized to the CTL group, is represented as a histogram **(E)**. Bars represent the mean ± SEM of all samples (*n* = 10 animals per group). Asterisks represent statistically significant differences. ***p < 0.001; ****p < 0.0001. Statistical test: ANOVA followed by Holm-Sidak *post hoc* test.

Similar observations were made when studying the OP (Figure 3). PA-induced a strong loss of complexity (*p* < 0.0001) in the OP patterns (Figures 3C,E) whereas MB treatment significantly (*p* < 0.001) restored the control pattern (Figures 3D,E).

### MB Treatment Reduces Asphyxia-Induced Thickening of the Inner Retina and GFAP Immunoreactivity

**Figure 4:**
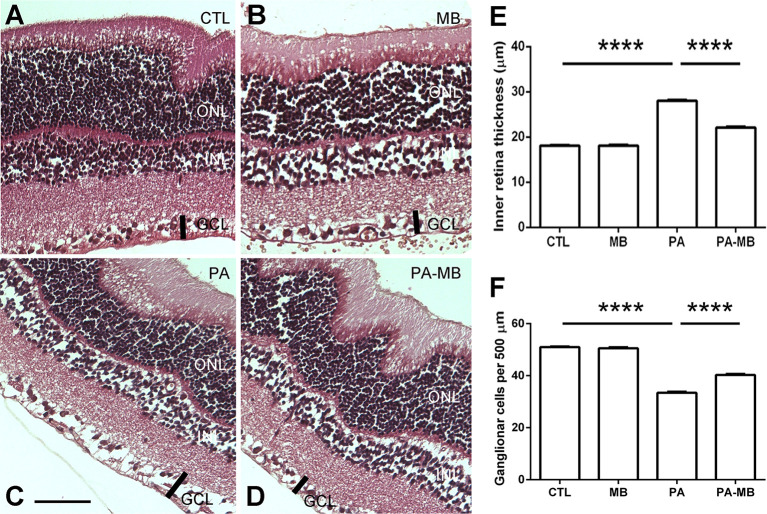
Representative histological images of the retina of animals of the four experimental groups: CTL **(A)** MB **(B)**, PA **(C)**, and PA-MB **(D)** taken 45 days after birth. Three layers of the retina are labeled in the pictures for reference: outer nuclear layer (ONL), inner nuclear layer (INL), and ganglion cell layer (GCL). The thick black bar demarcates the inner retina (IR). Horizontal bar = 50 μm. Quantification of the IR thickness **(E)** and the number of ganglion cells per 500 μm **(F)** are shown as histograms. Bars represent the mean ± SEM of all samples (*n* = 4 animals per group, five measurements per animal). Asterisks represent statistically significant differences, ****p < 0.0001. Statistical test: ANOVA followed by Holm-Sidak *post hoc* test.

It has been previously reported that PA results in morphological changes of the retina, including a thicker inner retina (IR), and an increased number of GFAP-positive cellular processes (Rey-Funes et al., 2016). This was also the case in the present study. The eyes of the PA group had a significantly (*p* < 0.0001) thicker IR (Figures 4C,E) than those of the CTL group (Figures 4A,E). Treatment of the control newborns with MB did not change this parameter (Figures 4B,E) but the treatment of the asphyctic pups significantly (*p* < 0.0001) prevented this morphological manifestation of the pathology (Figures 4D,E). The thickness of all other layers of the retina was measured and compared but no statistically significant differences were found among experimental groups (results not shown). The number of ganglion cells in a specified length (500 μm) was also compared among experimental groups (Figure 4F). There was a very significant reduction in the number of ganglion cells in the PA animals (*p* < 0.0001) compared to the control groups. In the PA-MB group, the number of ganglion cells was significantly higher (*p* < 0.0001) than in the PA group (Figure 4F).

**Figure 5:**
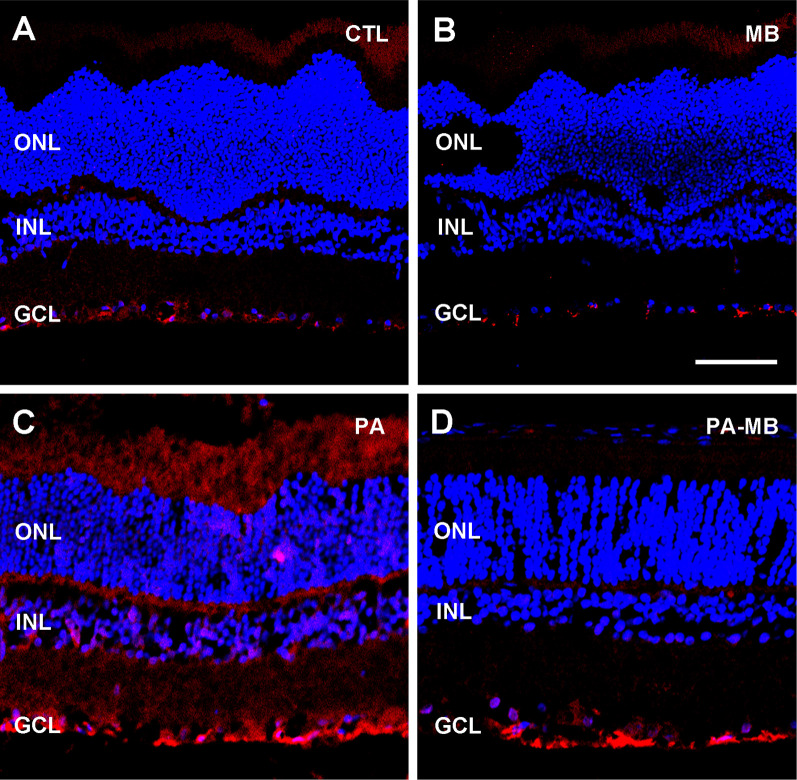
Representative confocal microscopy images, labeled for GFAP (red), of the retina of animals of the four experimental groups: CTL **(A)**, MB **(B)**, PA **(C)**, and PA-MB **(D)**, taken 45 days after birth. Three layers of the retina are labeled in the pictures for reference: ONL, INL, and GCL. Nuclei are counterstained with DAPI (blue). GFAP staining is more intense in the PA group than in all the others. Horizontal bar = 50 μm.

Furthermore, GFAP immunostaining showed a marked increase in GFAP signal in the PA group (Figure 5C) compared with either the CTL (Figure 5A) or MB (Figure 5B) groups. Application of MB (Figure 5D) reduced GFAP expression to levels similar to those observed in the controls.

### MB Treatment Reduces Asphyxia-Induced Apoptosis in the Ganglion Cell Layer

**Figure 6:**
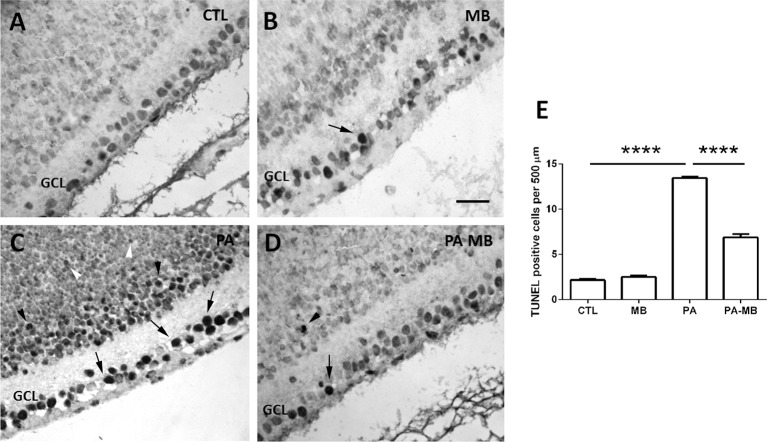
TUNEL positive cells in the four experimental groups 6 days post-treatment. Representative images of retinas from CTL **(A)**, MB **(B)**, PA **(C)**, and PA-MB **(D)** animals. TUNEL positive cells were found mainly in the GCL (arrows), in the precursors of the INL (black arrowheads), and of the ONL (white arrowheads). Bar = 20 μm. Quantification of the results is shown as a histogram **(E)**. Bars represent the mean ± SEM of all samples (*n* = 4 animals per group, five measurements per animal). Asterisks represent statistically significant differences, ****p < 0.0001. Statistical test: ANOVA followed by Holm-Sidak *post hoc* test.

TUNEL analysis of the retinas (Figure 6) showed that exposure to PA results in a very significant (*p* < 0.0001) increase in the number of apoptotic cells (arrows) in the GCL (Figures 6C,E), whereas control animals have a very low number of labeled cells (Figures 6A,E). Postnatal treatment with MB significantly (*p* < 0.0001) reduced the number of apoptotic cells in the retina of asphyctic animals (Figures 6D,E) whereas it had no significant effect on non-asphyctic retinas (Figures 6B,E). A large number of apoptotic cells was observed among the neuroblasts that would develop into the inner (black arrowheads in Figure 6C) and outer (white arrowheads in Figure 6C) nuclear layers of the retina of PA animals. These were not seen in control animals (Figures 6A,B) and were very scarce in MB-treated rats (Figure 6D).

**Figure 7:**
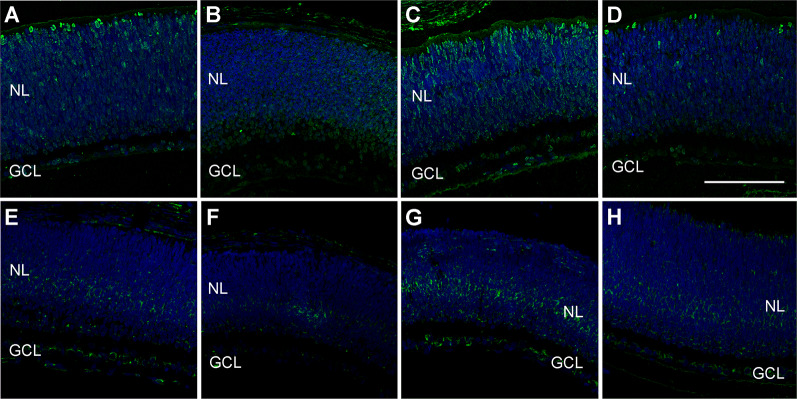
Representative confocal microscopy images, labeled in green for HNEJ-2 **(A–D)** or 8-OHdG **(E–H)**, of the retina of animals of the four experimental groups: CTL **(A,E)**, MB **(B,F)**, PA **(C,G)**, and PA-MB **(D,H)**, taken 6 days after birth. Two layers of the retina are labeled in the pictures for reference: nuclear layer (NL; animals are too young to distinguish the ONL and the INL yet), and GCL. Nuclei are counterstained with DAPI (blue). Increased staining is found for both HNEJ-2 and 8-OHdG in the PA samples compared to the other groups. Horizontal bar = 100 μm.

**Figure 8:**
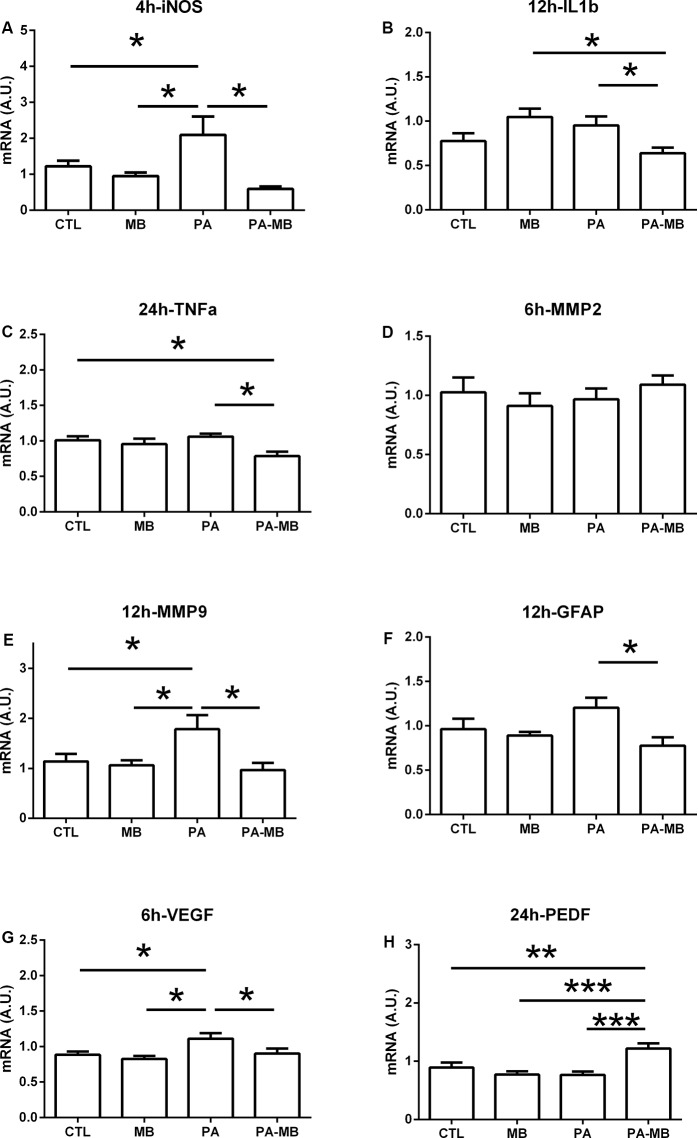
Relative gene expression in the retina of the four experimental groups for iNOS **(A)**, IL1β **(B)**, TNFα **(C)**, MMP2 **(D)**, MMP9 **(E)**, GFAP **(F)**, VEGF **(G)**, and PEDF **(H)**. Samples were collected at the indicated times after treatment. All data represent the quotient between the gene of interest and the expression of the housekeeping gene 18S. Bars represent the mean ± SEM of all measurements (*n* = 6–8). Asterisks represent statistically significant differences as indicated. *p < 0.05, **p < 0.01; ***p < 0.001. Statistical test: ANOVA followed by the Newman–Keuls *post hoc* test.

Also, retina sections were exposed to antibodies against 4-hydroxynonenal (HNEJ-2; Figures 7A–D) and 8-hydroxy-2’-deoxyguanosine (8-OHdG; Figures 7E–H) to further study tissue damage due to asphyxia. For both markers, there was a signal increase in the retinas of the PA group (Figures 7C,G) when compared to the CTL (Figures 7A,E) and the MB group (Figures 7B,F). Also, in both cases, the application of MB post-asphyxia resulted in a decrease in the damage markers (Figures 7D,H).

### MB Treatment Modulates Gene Expression of Key Players in Inflammation, Gliosis, Matrix Remodeling, and Angiogenesis

Gene expression was studied in the retina for several markers (Table 2) at different times after MB injection. There was a clear time-specific modulation that was different for each gene. The earliest significant change occurred for iNOS expression (Figure 8A). At 4 h after treatment, there was a significant (*p* < 0.05) increase of iNOS in the PA animals when compared to CTL and MB groups. Treatment of PA animals with MB completely prevented iNOS overexpression (*p* < 0.05). Other inflammation markers whose expression was modulated by MB included IL1β at 12 h (Figure 8B) and TNFα at 24 h (Figure 8C). In both cases, there was no significant increase of expression in the PA group over controls but there was a significant (*p* < 0.05) decrease in the PA-MB group when compared to the PA animals.

Matrix metalloproteinases (MMP) are key enzymes on tissue remodeling. We did not see any significant modulation of MMP2 at any time by either asphyxia or MB treatment (Figure 8D). On the other hand, MMP9 expression was significantly (*p* < 0.05) increased on the PA group at 12 h and this pathological increase was fully prevented (*p* < 0.05) by MB treatment (Figure 8E). Coinciding with these MMP9 changes, we also found a significant decrease of GFAP expression, a marker of gliosis, on the PA-MB group when compared to the PA animals at 12 h (Figure 8F).

Increased angiogenesis is a typical response to hypoxia/ischemia in the retina (Rey-Funes et al., 2013) and we studied the expression of a positive regulator, VEGF, and a negative regulator of angiogenesis, PEDF. At 6 h there was a significant (*p* < 0.05) increase of VEGF expression in the retinas of asphyctic rats, and this increase was significantly counteracted (*p* < 0.05) by MB treatment (Figure 8G). Also, although the expression of PEDF did not change in the PA group, there was a very significant (*p* < 0.001) increase in the PA-MB group when compared to all other experimental groups (Figure 8H). This PEDF behavior was observed both at 6 h and at 24 h.

## Discussion

In this study, we have shown that MB applied to newborns that have suffered PA has very significant advantages in retinal electrophysiology, morphological markers of retinal pathology, and gene expression modulation, suggesting that this treatment may be useful in preventing retinal damage and visual loss associated to PA.

This study follows our previous work where we showed that MB injected into pregnant dams before delivery had a beneficial impact on the retinal health of the newborns (Rey-Funes et al., 2016). However, although the risks for developing PA are well known (Martinez-Biarge et al., 2013; Bogdanovic et al., 2014), it is difficult to convince the mothers and the medical staff to apply a preventative treatment just before delivery. Our current data show that MB treatment is, at least, as efficient when applied to newborns a few hours after delivery, once the asphyxia episode has occurred and it is evident to everyone involved that an intervention is needed.

Scotopic ERG showed that PA resulted in a significant reduction of the amplitude for both a- and b-waves, as well as a loss of complexity in the OP patterns. It is recognized that the a-wave is generated by the photoreceptors, the OP by the cells in the inner nuclear layer (INL), and the b-wave by the ganglion cells (Jung et al., 2015; Matei et al., 2020; Zhai et al., 2020). Different animal models of retinal damage influence ERG in a model-specific fashion. For instance, models of blunt ocular trauma (Blanch et al., 2014) or chemical intoxication (Chen et al., 2013) induce photoreceptor apoptosis and a-wave disruption. Conversely, ischemic retinopathy models (Osborne et al., 2004) or optic nerve injury (Rey-Funes et al., 2017) are characterized by impaired inner retinal function, showing changes in the b-wave. Therefore, we can conclude that PA affects all the components of the retinal visual axis. This was confirmed by the observation of TUNEL-positive cells in the three anatomical demarcations in the retina and overexpression of the damage markers NHEJ-2 and 8-OHdG in rats exposed to PA. Interestingly, the application of MB was able to normalize the physiological patterns as well as the morphological telltales of retinal pathology, indicating that this could constitute a new treatment to prevent visual loss in children affected by PA.

A common morphological feature observed in all models of ocular hypoxia/ischemia-reperfusion is the thickening of the inner layer of the retina due to a pathological increase in gliosis and angiogenesis (Rey-Funes et al., 2013; Luo et al., 2018). Gliosis is due to a proliferation of the GFAP-positive processes of Müller cells (Pekny et al., 2014) whereas the excessive number of blood vessels reflects a lost balance between proangiogenic and antiangiogenic factors within the eye environment (Friedlander, 2007). In the present study, both the immunoreactivity and RNA expression of GFAP was significantly downregulated by the treatment with MB. The proliferation of reactive astrocytes, or Müller cells in the case of the retina, is considered an obstacle for the proper physiological communication among neighboring neurons (Pekny et al., 2014), so we can consider that it may contribute to the compromised electrophysiological recordings we found in PA animals and, perhaps, to the excessive apoptosis of ganglion cells detected by direct counting and corroborated by TUNEL. The contribution of MB treatment to the reduction of retinal gliosis can thus be considered a very desirable outcome.

Our initial hypothesis was that MB can modulate NO production and function in the eye and, as a consequence, could provide neuroprotection for ischemic injuries of the retina, including PA. Our qRT-PCR results confirm this hypothesis and show a fast expression of the inducible form of NOS just 4 h after PA, which was prevented by MB. iNOS is a Ca^2+^ independent isoform of NOS whose expression rises rapidly in response to inflammatory signals and other injuries, including ischemia-reperfusion of the retina (Rodrigo et al., 2005). iNOS produces large amounts of NO which acts as a free radical and contributes to tissue damage (Toda and Nakanishi-Toda, 2007). The complete blocking of iNOS induction by MB treatment points to a mechanistic explanation for the beneficial effects of MB on preserving retinal function even after suffering the ischemic insult. Other inflammatory mediators that are regulated by MB are IL1β and TNFα. The mechanism by which MB reduces expression of these inflammatory markers is unknown but it may follow the earlier lowering on iNOS expression and a general reduction on the inflammatory milieu of the eye.

Eye angiogenesis must be very tightly controlled to avoid pathological blood vessel overproduction, which is the cause of many retinal and choroidal chronic diseases (Lau et al., 2018). The main proangiogenic factor of the eye is VEGF (Virgili et al., 2018) whereas the major antiangiogenic factor is PEDF (Farnoodian et al., 2017). Furthermore, there is a mutually opposite regulation between these two factors: VEGF can induce MMP expression, which in turn will degrade retinal PEDF (Notari et al., 2005). In our analysis, we found a fast upregulation of VEGF expression within 6 h of PA. This was to be expected since VEGF is transactivated by HIF-1 transcription factor, which rapidly signals to the nucleus under hypoxic conditions (Kurihara et al., 2014). That this upregulation was prevented by MB treatment suggests a preventive antiangiogenic effect for MB in the retina. Also, PEDF did not change in the PA animals but its expression was very significantly elevated in the PA animals that were treated with MB. This elevation of an antiangiogenic molecule, together with the downregulation of VEGF, may partly explain the protective effect of MB on retinal morphology and physiology.

Another aspect needed for angiogenesis and retinal thickening is extracellular matrix remodeling. Dynamic changes in the connective tissue depend on the activity of many proteases, including MMPs and, in particular, gelatinases (Jabłońska-Trypuć et al., 2016). Interestingly, MMP2 did not respond to either PA or the treatment with MB but MMP9 expression increased following PA. It has been shown that hypoxia (Li and Zheng, 2017) and iNOS (Anavi et al., 2015) can upregulate MMP9 expression under some conditions, and that MMP9 is a proangiogenic factor (Djordjevic et al., 2018), so the increased expression of MMP9 following PA may contribute to enhanced angiogenesis and the thickening of the IR we observed in these animals. Also, MMP9 has been implicated in the degradation of PEDF in the retina (Notari et al., 2005), thus further implicating this protease in angiogenesis regulation. The fact that MB was able to completely prevent MMP9 overexpression, probably through blockade of excessive NO availability, further supports the protective role of this chemical.

In summary, MB represents an effective treatment to reduce the physiological, morphological, and molecular telltales of retinal degeneration following episodes of PA and, given its safety profile and low cost, it could be used as an alternative therapy to hypothermia in regions of the world where that intervention may be unavailable.

## Data Availability Statement

The raw data supporting the conclusions of this article will be made available by the authors, without undue reservation, to any qualified researcher.

## Ethics Statement

The animal study was reviewed and approved by Ethical Committee of CICUAL (Comité Institucional para el Uso y Cuidado de Animales de Laboratorio).

## Author Contributions

JF, RP, MR-F, MS, DC, VD, and JL-C: acquisition, analysis, and interpretation of data. IML, CFL, and AM: conception and design, analysis and interpretation of data. AM: wrote the article. All authors revised the original manuscript and agreed on its contents.

## Conflict of Interest

The authors declare that the research was conducted in the absence of any commercial or financial relationships that could be construed as a potential conflict of interest.
